# Prognostic impact of prior LVEF in patients with heart failure with mildly reduced ejection fraction

**DOI:** 10.1007/s00392-024-02443-0

**Published:** 2024-04-15

**Authors:** Alexander Schmitt, Michael Behnes, Kathrin Weidner, Mohammad Abumayyaleh, Marielen Reinhardt, Noah Abel, Felix Lau, Jan Forner, Mohamed Ayoub, Kambis Mashayekhi, Ibrahim Akin, Tobias Schupp

**Affiliations:** 1 First Department of Medicine, Section for Invasive Cardiology, University Medical Centre Mannheim (UMM), Medical Faculty Mannheim, Heidelberg University, Theodor-Kutzer-Ufer 1-3, 68167 Mannheim, Germany; 2 Division of Cardiology and Angiology, Heart Centre University of Bochum, Bad Oeynhausen, Germany; 3 Department of Internal Medicine and Cardiology, Mediclin Heart Centre Lahr, Lahr, Germany

**Keywords:** Heart failure with mildly reduced ejection fraction, HFmrEF, LVEF, Prognosis, Mortality, longitudinal changes of LVEF

## Abstract

**Aims:**

As there is limited evidence regarding the prognostic impact of prior left ventricular ejection fraction (LVEF) in patients with heart failure with mildly reduced ejection fraction (HFmrEF), this study investigates the prognostic impact of longitudinal changes in LVEF in patients with HFmrEF.

**Methods:**

Consecutive patients with HFmrEF (i.e. LVEF 41–49% with signs and/or symptoms of HF) were included retrospectively in a monocentric registry from 2016 to 2022. Based on prior LVEF, patients were categorized into three groups: stable LVEF, improved LVEF, and deteriorated LVEF. The primary endpoint was 30-months all-cause mortality (median follow-up). Secondary endpoints included in-hospital and 12-months all-cause mortality, as well as HF-related rehospitalization at 12 and 30 months. Kaplan–Meier and multivariable Cox proportional regression analyses were applied for statistics.

**Results:**

Six hundred eighty-nine patients with HFmrEF were included. Compared to their prior LVEF, 24%, 12%, and 64% had stable, improved, and deteriorated LVEF, respectively. None of the three LVEF groups was associated with all-cause mortality at 12 (*p* ≥ 0.583) and 30 months (31% vs. 37% vs. 34%; log rank *p* ≥ 0.376). In addition, similar rates of 12- (*p* ≥ 0.533) and 30-months HF-related rehospitalization (21% vs. 23% vs. 21%; log rank *p* ≥ 0.749) were observed. These findings were confirmed in multivariable regression analyses in the entire study cohort.

**Conclusion:**

The transition from HFrEF and HFpEF towards HFmrEF is very common. However, prior LVEF was not associated with prognosis, likely due to the persistently high dynamic nature of LVEF in the follow-up period.

**Graphical Abstract:**

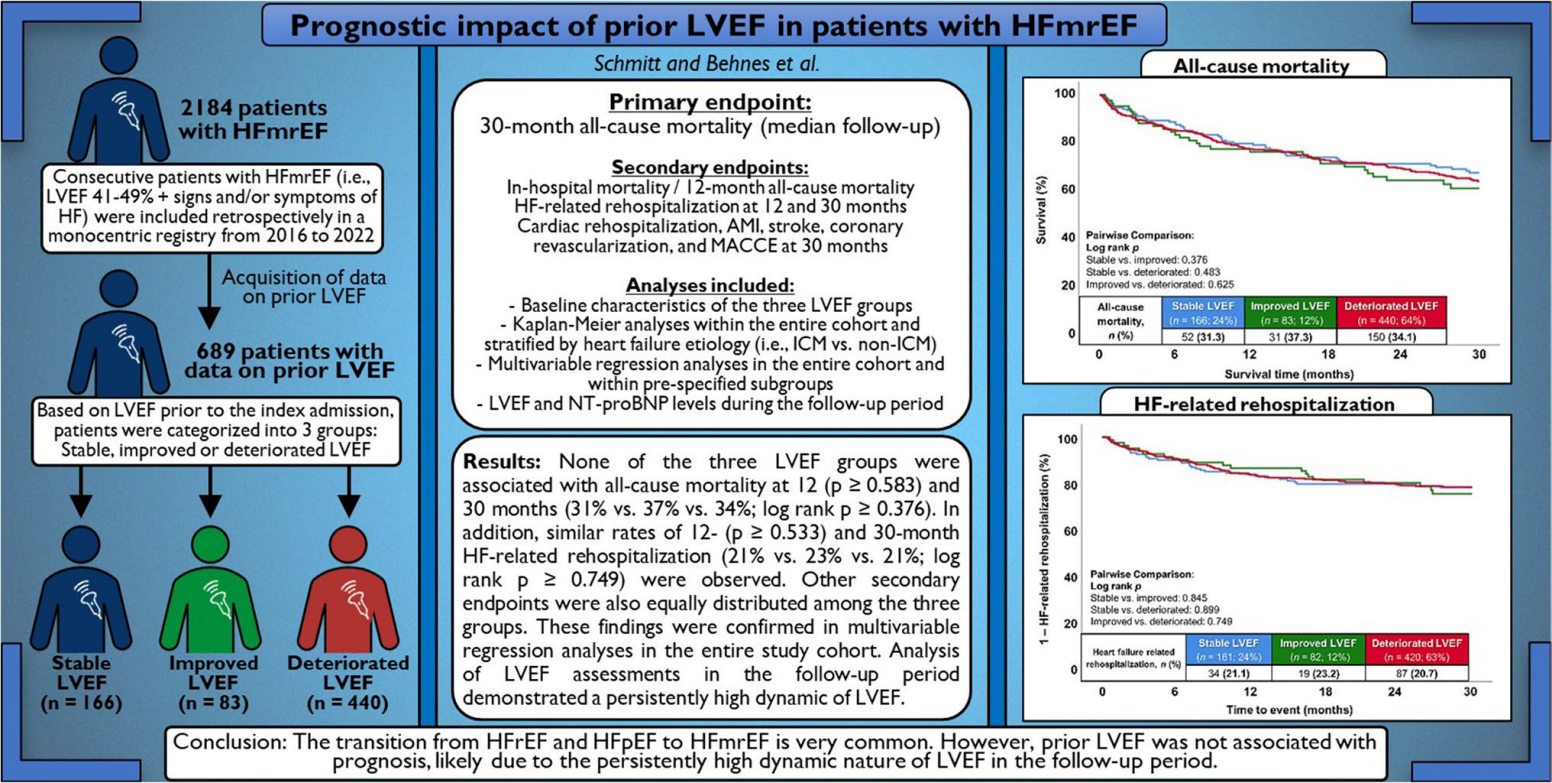

**Supplementary Information:**

The online version contains supplementary material available at 10.1007/s00392-024-02443-0.

## Introduction

Within the last decades, the prevalence of heart failure (HF) has steadily increased due to ongoing demographic changes related to an overall ageing population [1, 2]. Global estimates have shown that approximately 64 million people are affected by HF and data from the United States suggests that total health care expenditure for the management of HF could rise to 70 billion US-dollars by 2030 [3, 4]. Even though HF can be described through a variety of parameters, clinical signs, or symptomatology, it is most commonly classified by left ventricular ejection fraction (LVEF) [5, 6]. Until recently, patients were either divided into the category of HF with reduced (HFrEF) or preserved ejection fraction (HFpEF). However, within the past years, the European and American HF guidelines have introduced an additional category of HF with an LVEF of 41–49%, the so-called heart failure with mildly reduced ejection fraction (HFmrEF) [5, 6]. This category accounts for 16–24% of patients with HF [7, 8]. Furthermore, the category of HF with improved ejection fraction (HFimpEF) has been defined in a widely accepted position paper on the universal definition and classification of HF [9]. This classification aims to consider the potential prognostic implications of longitudinal changes of LVEF. According to this position paper, HFimpEF should be defined by a baseline LVEF ≤ 40% and a second measurement of LVEF > 40% with a ≥ 10% increase from baseline LVEF. Recent evidence from the ESC Heart Failure Long-Term Registry suggests that despite improvements in medical management, mortality of HF remains high with 1-year mortality rates of 8.8%, 7.8%, and 6.4% for HFrEF, HFmrEF, and HFpEF, respectively [7]. Within the same registry, Chioncel et al*.* were able to demonstrate a nearly linear increase in mortality and HF-related rehospitalization across every decile of reduced LVEF [7, 10]. However, since LVEF is a dynamic parameter, prognostic implications of LVEF changes over time must be considered to properly guide medical management of patients. As previous studies have demonstrated, improvement of LVEF in patients suffering from HFrEF is associated with favourable outcomes compared to patients with persistently reduced LVEF and maybe even those with stable HFpEF [11, 12]. Accordingly, deterioration of LVEF over time was observed to coincide with a worse prognosis [13–15]. Despite the importance of longitudinal changes in LVEF, there is limited evidence regarding the prognostic impact of prior LVEF in patients with HFmrEF. Since the category of HFmrEF was recently introduced, evidence guiding clinical decision-making for this cohort remains limited and the few guideline recommendations currently available are predominantly based on post hoc analyses of prior trials enrolling patients within the LVEF range of HFmrEF (e.g. CHARM-Preserved [16], TOPCAT [17], or PARAGON-HF [18]).

Therefore, the present study investigates the prognostic impact of prior LVEF in consecutive patients hospitalized with HFmrEF within a large-scaled retrospective registry-based analysis.

## Methods

### Study patients, design, and data collection

For the present study, all consecutive patients hospitalized with HFmrEF at one University Medical Centre were included from January 2016 to December 2022, as recently published [19]. Using the electronic hospital information system, all relevant clinical data related to the index event were documented, such as baseline characteristics; vital signs on admission; prior medical history; prior medical treatment; length of index hospital and intensive care unit (ICU) stay; laboratory values; data derived from all non-invasive or invasive cardiac diagnostics and device therapies, such as echocardiographic data, coronary angiography, and data being derived from prior or newly implanted cardiac devices. Every re-visit at the outpatient clinic or rehospitalizations related to HF or adverse cardiac events were documented until the end of the year 2022.

The present study is derived from the “Heart Failure With Mildly Reduced Ejection Fraction Registry” (HARMER), representing a retrospective single-centre registry including consecutive patients with HFmrEF hospitalized at the University Medical Centre Mannheim (UMM), Germany (clinicaltrials.gov identifier, NCT05603390). The registry was carried out according to the principles of the Declaration of Helsinki and was approved by the medical ethics committee II of the Medical Faculty Mannheim, University of Heidelberg, Germany (ethical approval code, 2022–818).

### Inclusion and exclusion criteria

All consecutive patients ≥ 18 years of age hospitalized with HFmrEF at one institution were included. Patients without echocardiographic assessment of LVEF prior to the index hospitalization were excluded. Furthermore, patients with a prior LVEF ≤ 40% but an LVEF improvement < 10% were excluded [9]. All included patients underwent at least one standardized transthoracic echocardiography at the cardiologic department at index hospitalization, where the diagnosis of HFmrEF was assessed. The diagnosis of HFmrEF was determined according to the “2021 European Society of Cardiology (ESC) Guidelines for the diagnosis and treatment of acute and chronic HF” [20]. Accordingly, all patients with an LVEF of 41–49% and symptoms and/or signs of HF were included. The presence of elevated amino-terminal prohormone of brain natriuretic peptide (NT-proBNP) levels and other evidence of structural heart disease were considered to make the diagnosis more likely but were not mandatory for the diagnosis of HFmrEF. Transthoracic echocardiography was exclusively performed by attending cardiologists or under their direct supervision in clinically stable patients and in accordance with current European guidelines [21] to ensure high standards of echocardiographic examination. The corresponding cardiologists were blinded to the final study analysis. LVEF was routinely measured by the established biplane method of disks summation (modified Simpson’s rule) recommended by the American Society of Echocardiography and the European Association of Cardiovascular Imaging [22]. All echocardiographic examinations and reports of the index admission were re-assessed post hoc by two independent cardiologists. In cases of ambiguous findings or documentation, echocardiographic source data was re-assessed based on the available Digital Imaging and Communications in Medicine (DICOM) files. Information on LVEF before the index admission was obtained through comprehensive investigation of all patient-related documents stored in the electronic hospital information system, including previous visits at our University Medical Centre Mannheim and available medical reports of other clinics or outpatient cardiology visits. In accordance with the index echocardiography, data on prior LVEF was only ascertained from documented transthoracic echocardiographic examinations measuring LVEF with the modified Simpson’s rule. To clarify, data on LVEF during the index admission was solely ascertained from inpatients, whereas data on prior LVEF was derived from patients in both the inpatient and outpatient settings.

### Risk stratification

For the present study, risk stratification was performed according to longitudinal changes of LVEF, and patients were divided into the following three groups in line with the universal definition and classification of HF [9]: stable LVEF (i.e. prior LVEF of 41–49%), improved LVEF (i.e. prior LVEF ≤ 40% and an improvement ≥ 10% compared to the prior LVEF), and deteriorated LVEF (i.e. prior LVEF ≥ 50%). The value of prior LVEF used for the categorization of patients into the three groups (i.e. stable, improved, and deteriorated) was defined as the most recent available LVEF assessment preceding the index hospitalization. No maximum time interval between the index admission and the previous echocardiographic assessment of prior LVEF was applied for the main analyses. Subanalyses with a minimum time interval of 1 month and a maximum time interval of 12 as well as 24 months between the prior and index echocardiography were performed. Furthermore, prognosis of patients with stable, improved, and deteriorated LVEF was also investigated stratified by the etiology of heart failure (i.e. ischemic and non-ischemic cardiomyopathy). Ischemic cardiomyopathy (ICM) comprised patients with prior documented coronary artery disease (CAD) or newly diagnosed CAD assessed by coronary angiography at the index hospitalization sufficient to cause myocardial dysfunction. Identification of CAD (i.e. at least one relevant stenosis of one epicardial coronary artery of more than 50%) was based on the judgment of the investigating interventional cardiologist during routine care. All coronary angiograms and reports were re-assessed post hoc by two independent interventional cardiologists to determine whether the CAD is sufficient for causality of myocardial dysfunction [23]. The group of non-ischemic cardiomyopathy (NICM) comprised all patients with other etiologies of heart failure as listed in Table 2 (i.e. primary non-ischemic cardiomyopathy [23], hypertensive cardiomyopathy [24], congenital heart disease, valvular heart disease [25, 26], tachycardia-induced [27], and pacemaker-induced cardiomyopathy [28]) excluding patients with unknown etiology. The parameter of right ventricular dysfunction (RVD) used in multivariable analyses was defined as a tricuspid annular plane systolic excursion (TAPSE) of < 17 mm. In patients with a history of cardiac surgery, systolic velocity of the tricuspid annulus (*S*′) < 9.5 cm/s was additionally considered to confirm RVD.

### Study endpoints

The primary endpoint was long-term all-cause mortality. Long-term was defined as the median time of clinical follow-up in months (i.e. 30 months). Secondary endpoints comprised in-hospital all-cause mortality, all-cause mortality at 12 months, rehospitalization for worsening HF at 12 and 30 months as well as cardiac rehospitalization, acute myocardial infarction (AMI), stroke, coronary revascularization, and major adverse cardiac and cerebrovascular events (MACCE) at long-term follow-up as well as changes in LVEF and NT-pro BNP levels during the follow-up period. All-cause mortality was documented using the electronic hospital information system and by directly contacting state resident registration offices (“bureau of mortality statistics”). HF-related hospitalization was defined as a rehospitalization due to worsening HF requiring intravenous diuretic therapy. HF-related rehospitalization comprised patients with hospitalization due to worsening HF as the primary cause or as a result of another cause but associated with worsening HF at the time of admission, or as a result of another cause but complicated by worsening HF during its cause. Cardiac rehospitalization was defined as rehospitalization due to a primary cardiac condition, including worsening HF, AMI, coronary revascularization, and symptomatic atrial or ventricular arrhythmias. MACCE was defined as the composite of all-cause mortality, coronary revascularization, non-fatal AMI, and non-fatal stroke. Time-trend subanalyses evaluated the course of LVEF and NT-proBNP serum levels at follow-up every 6 months in patients assigned to the stable, improved, and deteriorated LVEF groups. Here, all available echocardiographic examinations being investigated during routine care either within (re-)hospitalization or in the outpatient clinic at our institution were documented at the intervals of 0–6, 6–12, 12–18, 18–24, and 24–30 months. Assessments of dynamic transitions between LVEF-based HF categories were only presented for the 12 months following the index admission due to the limited number of consecutive echocardiographic LVEF assessments in patients during the follow-up period.

### Statistical methods

Quantitative data is presented as mean ± standard error of mean (SEM) or median with IQR, depending on the distribution of the data. They were compared using Student’s *t*-test for normally distributed data or the Mann–Whitney *U* test for nonparametric data. Deviations from a Gaussian distribution were tested by the Kolmogorov–Smirnov test. Qualitative data is presented as absolute and relative frequencies and were compared using the chi-square test or Fisher’s exact test, as appropriate. Kaplan–Meier analyses were performed stratified by the three LVEF groups (i.e. stable, improved, and deteriorated). Univariable hazard ratios (HR) were given together with 95% confidence intervals (CI). The prognostic impact of stable, improved, and deteriorated LVEF was thereafter investigated within multivariable Cox regression models. Multivariable Cox regression analyses were performed within the entire study cohort, as well as in pre-specified subgroups stratified by ≥ 75 and < 75 years of age, sex, acute decompensated heart failure (ADHF) in the index admission, and HF etiology as well as medical therapy at discharge. Multivariable Cox regression analyses were visualized using forest plots. LVEF and NT-pro BNP levels were compared among patients stratified by stable, improved, and deteriorated LVEF within 6-months intervals following the index hospitalization using Student’s *t*-test or the Mann–Whitney *U* Test.

Results of all statistical tests were considered significant for *p* ≤ 0.05. SPSS (Version 28, IBM, Armonk, NY) was used for statistics.

## Results

### Study population

**Fig. 1:**
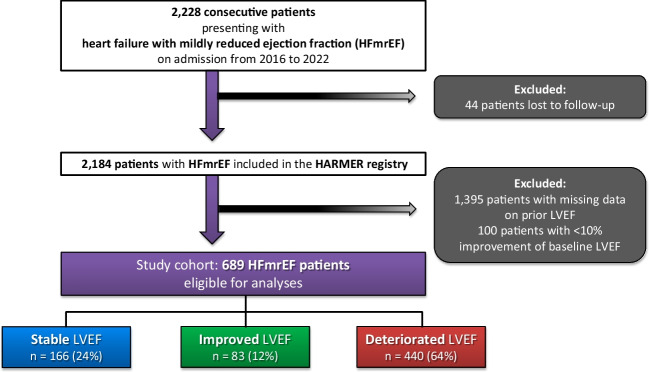
Study flow chart

From 2016 to 2022, 2228 patients hospitalized with HFmrEF were included in the HARMER registry. Of those, 44 patients with no evidence on long-term follow-up (corresponding lost-to follow-up rate, 1.97%), 1395 with no evidence on prior LVEF, and 100 patients with prior LVEF ≤ 40% but an improvement < 10% were excluded. Therefore, the final study cohort comprised 689 patients with a median duration between prior and index echocardiography of 308 days with an interquartile range (IQR) of 105–764 days. Within the entire study cohort, 166 (24%), 83 (12%), and 440 (64%) were assigned to the stable, improved, and deteriorated LVEF groups, respectively (Fig. 1; flow chart). Accordingly, longitudinal LVEF changes were very common in the HFmrEF cohort, with only 24% of patients remaining in the LVEF range of HFmrEF over time.

Table 1 displays baseline characteristics stratified by the three LVEF groups. Age and sex were equally distributed among the three groups (*p* ≥ 0.203 for all comparisons). Prior coronary artery disease (CAD) was most common among patients with stable LVEF (71.7%), but also highly prevalent in those with improved and deteriorated LVEF (66.3% and 57.5%, respectively). Accordingly, prevalence of prior myocardial infarction was significantly higher in the stable LVEF group when compared to the improved and deteriorated groups (48.2% and 34.9% vs. 33.4%; *p* ≤ 0.047 for both comparisons), which was reflected in the proportions of patients who had received percutaneous coronary intervention (PCI) in the past (57.2% and 45.8% vs. 43.0%). ADHF in the 12 months prior to the index admission was more common in the groups of stable and improved LVEF than in the group with deteriorated LVEF (30.7% and 39.8% vs. 18.0%; *p* ≤ 0.001 for both comparisons). Median LVEF determined prior to the index admission was 45%, 33%, and 60% in the groups with stable, improved, and deteriorated LVEF, respectively. In line with the guideline recommendations for the implantation of cardioverter defibrillators, the highest rates of implanted cardioverter defibrillators and cardiac resynchronization therapy with defibrillators were observed in patients with improved LVEF (i.e. prior LVEF ≤ 40% and an improvement ≥ 10% compared to the prior LVEF). There were no differences regarding prior comorbidities (i.e. chronic kidney disease or stroke) or most cardiovascular risk factors such as arterial hypertension, diabetes, or hyperlipidemia between all three groups (*p* ≥ 0.05 for all comparisons). Comorbidities during the index hospitalization were also equally distributed with only minor differences among the three LVEF groups. Data on medical therapies on admission is provided in the lower section of Table 1.

**Table 1:** Baseline characteristics

	Stable LVEF (*n* = 166)		Improved LVEF (*n* = 83)		Deteriorated LVEF (*n* = 440)		*p*-value Stable vs. improved	*p*-value Stable vs. deteriorated	*p*-value Improved vs. deteriorated
Age, median (IQR)	75	(65–84)	76	(61–81)	77	(67–83)	0.414	0.715	0.203
Male sex, *n* (%)	109	(65.7)	54	(65.1)	276	(62.7)	0.925	0.503	0.686
Body mass index, kg/m^2^, median (IQR)	26.6	(24.1–30.9)	26.1	(23.2–30.8)	26.4	(23.8–30.5)	0.282	0.507	0.513
SBP, mmHg, median (IQR)	140	(120–166)	132	(112–155)	143	(125–163)	**0.050**	0.516	**0.008**
DBP, mmHg, median (IQR)	73	(65–86)	74	(63–81)	80	(69–90)	0.471	**0.024**	**0.008**
Heart rate, bpm, median (IQR)	78	(67–91)	75	(66–89)	80	(68–95)	0.528	0.100	0.063
Medical history, *n* (%)									
Coronary artery disease	119	(71.7)	55	(66.3)	253	(57.5)	0.379	**0.001**	0.137
Prior myocardial infarction	80	(48.2)	29	(34.9)	147	(33.4)	**0.047**	**0.001**	0.787
Prior PCI	95	(57.2)	38	(45.8)	189	(43.0)	0.088	**0.002**	0.633
Prior CABG	29	(17.5)	8	(9.6)	59	(13.4)	0.101	0.206	0.346
Prior valvular surgery	13	(7.8)	8	(9.6)	29	(6.6)	0.629	0.592	0.321
Duration of heart failure, months, median (IQR)	27.7	(7.0–57.0)	34.5	(5.8–80.0)	31.2	(8.5–66.6)	0.501	0.389	0.997
Decompensated heart failure < 12 months	51	(30.7)	33	(39.8)	79	(18.0)	0.155	**0.001**	**0.001**
*Prior LVEF, %, median (IQR)	45	(45–45)	33	(25–35)	60	(56–60)	**0.001**	**0.001**	**0.001**
*Prior NT-pro BNP, pg/mL	1289	(498–3470)	2034	(942–5567)	2232	(1013–4374)	0.198	0.246	0.698
Prior ICD	6	(3.6)	8	(9.6)	9	(2.0)	0.052	0.268	**0.001**
Prior sICD	1	(0.6)	3	(3.6)	3	(0.7)	0.075	0.914	**0.021**
Prior CRT-D	6	(3.6)	10	(12.0)	5	(1.1)	**0.011**	**0.042**	**0.001**
Prior pacemaker	24	(14.5)	12	(14.5)	53	(12.0)	1.000	0.426	0.541
Chronic kidney disease	66	(39.8)	35	(42.2)	195	(44.3)	0.715	0.312	0.717
Peripheral artery disease	26	(15.7)	14	(16.9)	76	(17.3)	0.807	0.637	0.929
Stroke	37	(22.3)	19	(22.9)	83	(18.9)	0.915	0.345	0.396
Liver cirrhosis	3	(1.8)	4	(4.8)	8	(1.8)	0.175	0.993	0.094
Malignancy	20	(12.0)	12	(14.5)	77	(17.5)	0.592	0.103	0.499
COPD	33	(19.9)	16	(19.3)	69	(15.7)	0.910	0.218	0.415
Cardiovascular risk factors, *n* (%)									
Arterial hypertension	139	(83.7)	72	(86.7)	374	(85.0)	0.533	0.700	0.680
Diabetes mellitus	65	(39.2)	25	(30.1)	156	(35.5)	0.162	0.398	0.349
Hyperlipidemia	72	(43.4)	38	(45.8)	171	(38.9)	0.718	0.312	0.238
Smoking									
Current	30	(18.1)	20	(24.1)	66	(15.0)	0.263	0.356	**0.040**
Former	43	(25.9)	13	(15.7)	101	(23.0)	0.068	0.447	0.140
Family history	26	(15.7)	5	(6.0)	41	(9.3)	**0.030**	**0.026**	0.331
Comorbidities at index hospitalization, *n* (%)									
Acute coronary syndrome									
Unstable angina	6	(3.6)	8	(9.6)	18	(4.1)	0.052	0.789	**0.033**
STEMI	5	(3.0)	0	(0.0)	19	(4.3)	0.110	0.462	0.054
NSTEMI	17	(10.2)	6	(7.2)	52	(11.8)	0.439	0.586	0.222
Acute decompensated heart failure	41	(24.7)	24	(28.9)	135	(30.7)	0.475	0.148	0.748
Cardiogenic shock	1	(0.6)	2	(2.4)	18	(4.1)	0.218	**0.028**	0.464
Atrial fibrillation	78	(47.0)	40	(48.2)	224	(50.9)	0.858	0.389	0.650
Cardiopulmonary resuscitation	2	(1.2)	2	(2.4)	13	(3.0)	0.476	0.216	0.785
Out-of-hospital	0	(0.0)	0	(0.0)	4	(0.9)	-	0.218	0.383
In-hospital	2	(1.2)	2	(2.4)	9	(2.0)	0.476	0.489	0.832
Stroke	10	(6.0)	10	(12.0)	33	(7.5)	0.099	0.528	0.166
Medication on admission, *n* (%)									
ACE-inhibitor	70	(42.2)	46	(55.4)	162	(36.8)	**0.048**	0.277	**0.001**
ARB	47	(28.3)	17	(20.5)	131	(29.8)	0.183	0.725	0.085
Beta-blocker	133	(80.1)	65	(78.3)	317	(72.0)	0.739	**0.043**	0.238
Aldosterone antagonist	30	(18.1)	29	(34.9)	41	(9.3)	**0.003**	**0.003**	**0.001**
ARNI	2	(1.2)	3	(3.6)	2	(0.5)	0.201	0.309	**0.007**
SGLT2-inhibitor	3	(1.8)	0	(0.0)	8	(1.8)	0.218	0.993	0.216
Loop diuretics	91	(54.8)	58	(69.9)	207	(47.0)	**0.022**	0.088	**0.001**
Statin	108	(65.1)	53	(63.9)	269	(61.1)	0.851	0.374	0.640
ASA	77	(46.4)	28	(33.7)	172	(39.1)	0.057	0.104	0.357
P2Y12-inhibitor	37	(22.3)	13	(15.7)	50	(11.4)	0.219	**0.001**	0.270
DOAC	61	(36.7)	32	(38.6)	148	(33.6)	0.781	0.472	0.387
Vitamin K antagonist	13	(7.8)	11	(13.3)	51	(11.6)	0.172	0.179	0.667

*ACE*, angiotensin-converting enzyme; *ARB*, angiotensin receptor blocker; *ARNI*, angiotensin receptor neprilysin inhibitor; *ASA*, acetylsalicylic acid; *CABG*, coronary artery bypass grafting; *CKD*, chronic kidney disease; *COPD*, chronic obstructive pulmonary disease; *CRT-D*, cardiac resynchronization therapy with defibrillator; *DBP*, diastolic blood pressure; *DOAC*, directly acting oral anticoagulant; *IQR*, interquartile range; *(N)STEMI*, non-ST-segment elevation myocardial infarction; *NT-pro BNP*, N-terminal prohormone of brain natriuretic peptide; *SBP*, systolic blood pressure; *SGLT2*, sodium glucose linked transporter 2; *(s)ICD*, (subcutaneous) implantable cardioverter defibrillator. Level of significance *p* ≤ 0.05. Bold type indicates statistical significance. *Data on prior LVEF and prior NT-pro BNP levels were obtained at the time of the previous echocardiographic examination before the index admission

Additional HF-related and procedural data is outlined in Table 2. Considering HF etiologies, the proportion of ICM was higher in patients with stable LVEF compared to those with improved LVEF and deteriorated LVEF (74.7% and 62.7% vs. 62.7%; *p* ≤ 0.049 for both comparisons). Additionally, hypertensive cardiomyopathy was more prevalent in the deteriorated compared to the stable and improved LVEF groups (7.7% vs. 1.8% and 2.4%). Echocardiographically measured left ventricular end-diastolic diameter (LVEDD) was higher in patients with stable and improved compared to deteriorated LVEF (50 mm and 52 mm vs. 48 mm; *p* ≤ 0.05 for both comparisons). Furthermore, diastolic dysfunction was most common in the deteriorated LVEF group (75.5%) and significantly more common than in the improved LVEF group (65.1; *p* = 0.048). Parameters regarding coronary angiography and baseline laboratory values were equally distributed between the stable, improved, and deteriorated LVEF groups. There were many differences in the medication at discharge, especially between the improved and deteriorated groups. Discharge prescription of angiotensin-converting enzyme inhibitors (59.8% vs. 44.8%; *p* = 0.013), aldosterone antagonists (30.5% vs. 14.5; *p* = 0.001), loop diuretics (74.4% vs. 57.1%; *p* = 0.004), and amiodarone (8.5% vs. 3.3%; *p* = 0.031) was more common in the improved compared to the deteriorated LVEF group. However, discharge prescription of angiotensin receptor blockers was more common in the deteriorated group (29.8% vs. 17.1%; *p* = 0.019).

**Table 2:** Heart failure–related and procedural data

	Stable LVEF (*n* = 166)		Improved LVEF (*n* = 83)		Deteriorated LVEF (*n* = 440)		*p*-value Stable vs. improved	*p*-value Stable vs. deteriorated	*p*-value Improved vs. deteriorated
Heart failure etiology, *n* (%)									
Ischemic cardiomyopathy	124	(74.7)	52	(62.7)	276	(62.7)	**0.049**	**0.006**	0.989
Non-ischemic cardiomyopathy	17	(10.2)	14	(16.9)	31	(7.0)	0.135	0.194	**0.003**
Hypertensive cardiomyopathy	3	(1.8)	2	(2.4)	34	(7.7)	0.749	**0.007**	0.079
Congenital heart disease	0	(0.0)	2	(2.4)	0	(0.0)	**0.045**	-	**0.001**
Valvular heart disease	8	(4.8)	5	(6.0)	28	(6.4)	0.687	0.473	0.907
Tachycardia associated	4	(2.4)	3	(3.6)	22	(5.0)	0.588	0.161	0.587
Tachymyopathy	2	(1.2)	2	(2.4)	4	(0.9)	0.476	0.743	0.239
Pacemaker-induced cardiomyopathy	1	(0.6)	0	(0.0)	3	(0.7)	0.479	0.914	0.451
Unknown	9	(5.4)	5	(6.0)	46	(10.5)	0.846	0.054	0.212
NYHA functional class, *n* (%)									
I/II	104	(62.7)	50	(60.2)	265	(60.2)	0.577	**0.010**	0.356
III	49	(29.5)	22	(26.5)	103	(23.4)			
IV	13	(7.8)	11	(13.3)	72	(16.4)			
Echocardiographic data									
LVEF, %, median (IQR)	45 (45–47)		45 (45–47)		45 (43–47)		0.541	0.719	0.660
IVSd, median (IQR)	12 (11–13)		11 (10–13)		12 (11–13)		**0.044**	0.530	0.071
LVEDD, mm, median (IQR)	50 (45–54)		52 (46–56)		48 (44–53)		0.308	**0.050**	**0.013**
TAPSE, mm, median (IQR)	20 (17–23)		19 (17–22)		19 (16–23)		0.136	0.137	0.647
LA diameter, mm, median (IQR)	42 (38–46)		44 (38–48)		43 (38–48)		0.706	0.688	0.978
LA surface, cm^2^, median (IQR)	21 (18–24)		24 (20–27)		23 (18–32)		**0.048**	0.075	0.569
* E*/*A*, median (IQR)	0.8 (0.6–1.3)		0.8 (0.5–1.1)		0.8 (0.6–1.2)		0.398	0.905	0.427
* E*/*E*′, median (IQR)	10.5 (6.0–14.5)		8.7 (4.5–13.3)		9.8 (6.0–14.0)		0.204	0.650	0.366
Diastolic dysfunction, *n* (%)	118	(71.1)	54	(65.1)	332	(75.5)	0.332	0.272	**0.048**
Moderate–severe aortic stenosis, *n* (%)	19	(11.4)	8	(9.6)	47	(10.7)	0.665	0.788	0.776
Moderate–severe aortic regurgitation, *n* (%)	8	(4.8)	6	(7.2)	22	(5.0)	0.436	0.927	0.408
Moderate–severe mitral regurgitation, *n* (%)	24	(14.5)	8	(9.6)	56	(12.7)	0.284	0.575	0.431
Moderate–severe tricuspid regurgitation, *n* (%)	27	(16.3)	20	(24.1)	86	(19.5)	0.137	0.355	0.344
VCI, mm, median (IQR)	21 (16–26)		22 (15–25)		22 (17–27)		0.989	0.617	0.615
Aortic root, mm, median (IQR)	33 (30–37)		34 (29–36)		33 (30–36)		0.665	0.346	0.767
Coronary angiography, *n* (%)	59	(35.5)	26	(31.3)	182	(41.4)	0.508	0.192	0.087
No evidence of coronary artery disease	8	(13.6)	7	(26.9)	31	(17.0)	0.304	0.174	0.309
1-vessel disease	6	(10.2)	1	(3.8)	29	(15.9)			
2-vessel disease	10	(16.9)	6	(23.1)	44	(24.2)			
3-vessel disease	35	(59.3)	12	(46.2)	78	(42.9)			
CABG	11	(18.6)	3	(11.5)	22	(12.1)	0.416	0.203	0.936
Chronic total occlusion	11	(18.6)	3	(11.5)	27	(14.8)	0.416	0.485	0.654
PCI	30	(50.8)	14	(53.8)	86	(47.3)	0.799	0.631	0.529
Sent to CABG	0	(0.0)	1	(3.8)	6	(3.3)	0.130	0.158	0.884
Baseline laboratory values, median (IQR)									
Potassium, mmol/L	3.9 (3.6–4.3)		3.9 (3.6–4.4)		3.9 (3.7–4.2)		0.697	0.693	0.477
Sodium, mmol/L	139 (137–141)		139 (137–142)		139 (137–141)		0.843	0.508	0.819
Creatinine, mg/dL	1.14 (0.90–1.64)		1.20 (0.92–1.64)		1.17 (0.94–1.67)		0.685	0.514	0.941
eGFR, mL/min/1.73 m2	62 (39–81)		59 (39–75)		57 (37–78)		0.663	0.329	0.783
Hemoglobin, g/dL	12.0 (9.7–13.6)		11.5 (9.5–13.5)		12.2 (10.1–13.6)		0.638	0.256	0.151
WBC count, × 10^9^/L	8.25 (6.28–10.16)		7.82 (6.02–9.15)		7.85 (6.19–9.79)		0.157	0.320	0.487
Platelet count, × 10^9^/L	217 (169–277)		237 (183–295)		223 (173–273)		0.135	0.496	0.236
HbA1c, %	5.8 (5.5–7.0)		5.7 (5.0–6.8)		5.9 (5.5–7.0)		0.468	0.761	0.347
LDL-cholesterol, mg/dL	88 (65–115)		82 (61–125)		89 (58–117)		0.442	0.881	0.357
HDL-cholesterol, mg/dL	42 (36–51)		41 (34–49)		44 (35–52)		0.867	0.520	0.507
C-reactive protein, mg/L	11 (3–38)		16 (4–39)		12 (3–45)		0.395	0.877	0.486
NT-pro BNP, pg/mL	2679 (1236–7153)		2447 (996–8882)		3558 (1650–9038)		0.769	0.329	0.285
Cardiac troponin I, µg/L	0.03 (0.02–0.16)		0.02 (0.02–0.15)		0.02 (0.02–0.10)		0.361	0.159	0.947
Medication at discharge, *n* (%)									
ACE-inhibitor	80	(49.7)	49	(59.8)	188	(44.8)	0.137	0.286	**0.013**
ARB	45	(28.0)	14	(17.1)	125	(29.8)	0.062	0.668	**0.019**
Beta-blocker	137	(85.1)	69	(84.1)	348	(82.9)	0.846	0.516	0.776
Aldosterone antagonist	40	(24.8)	25	(30.5)	61	(14.5)	0.347	**0.003**	**0.001**
ARNI	3	(1.9)	3	(3.7)	4	(1.0)	0.394	0.368	0.056
SGLT2-inhibitor	6	(3.7)	0	(0.0)	16	(3.8)	0.077	0.963	0.072
Loop diuretics	90	(55.9)	61	(74.4)	240	(57.1)	**0.005**	0.787	**0.004**
Statin	117	(72.7)	57	(69.5)	300	(71.4)	0.606	0.766	0.726
Digitalis	6	(3.7)	6	(7.3)	28	(6.7)	0.222	0.177	0.830
Amiodarone	6	(3.7)	7	(8.5)	14	(3.3)	0.115	0.816	**0.031**
ASA	81	(50.3)	32	(39.0)	190	(45.2)	0.095	0.273	0.300
P2Y12-inhibitor	56	(34.8)	22	(26.8)	124	(29.5)	0.209	0.220	0.623
DOAC	63	(39.1)	36	(43.9)	172	(41.0)	0.474	0.689	0.620
Vitamin K antagonist	14	(8.7)	7	(8.5)	39	(9.3)	0.967	0.825	0.830

*ACE*, angiotensin-converting enzyme; *ARB*, angiotensin receptor blocker; *ARNI*, angiotensin receptor neprilysin inhibitor; *ASA*, acetylsalicylic acid; *CABG*, coronary artery bypass grafting; *DOAC*, directly acting oral anticoagulant; *eGFR*, estimated glomerular filtration rate; *HbA1c*, glycated hemoglobin; *HDL*, high-density lipoprotein; *IQR*, interquartile range; *IVSd*, interventricular septum in diastole; *LA*, left atrial; *LDL*, low-density lipoprotein; *LVEDD*, left ventricular end-diastolic diameter; *LVEF*, left ventricular ejection fraction; *NT-pro BNP*, N-terminal prohormone of brain natriuretic peptide; *NYHA*, New York Heart Association; *PCI*, percutaneous coronary intervention; *TAPSE*, tricuspid annular plane systolic excursion; *VCI*, vena cava inferior; *WBC*, white blood cells. Level of significance *p* ≤ 0.05. Bold type indicates statistical significance

### Prognostic impact of prior LVEF in patients with HFmrEF

**Fig. 2:**
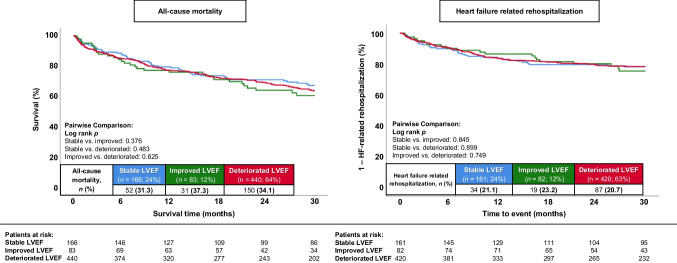
Kaplan–Meier analyses comparing patients with stable, improved, and deteriorated LVEF regarding the primary endpoint of long-term all-cause mortality (left panel) and secondary endpoint of long-term heart failure–related rehospitalization (right panel) within the entire study cohort

**Fig. 3:**
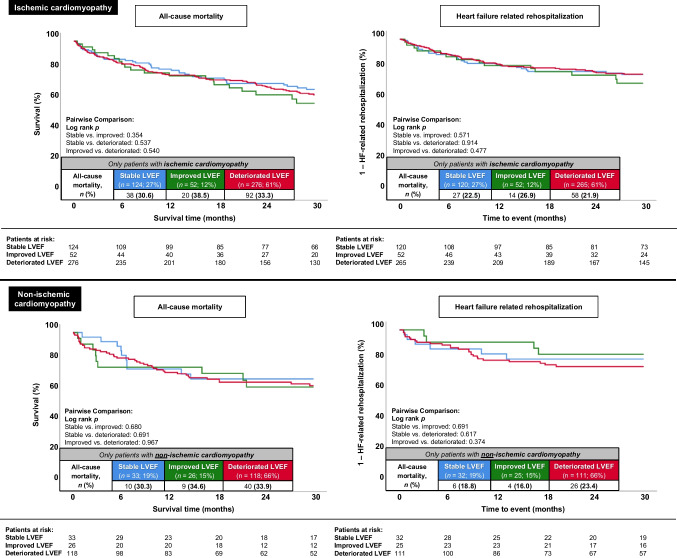
Kaplan–Meier analyses comparing patients with stable, improved, and deteriorated LVEF regarding the primary endpoint of long-term all-cause mortality (left panels) and secondary endpoint of long-term heart failure–related rehospitalization (right panels) within subgroups of patients with ischemic (upper panels) or non-ischemic cardiomyopathy (lower panels)

The development of LVEF over time was not significantly associated with the primary endpoint. Therefore, compared to the previously documented LVEF, patients with stable (HR = 0.880; 95% CI 0.646–1.198; *p* = 0.416), improved (HR = 1.133; 95% CI 0.776–1.653; *p* = 0.518), and deteriorated (HR = 1.041; 95% CI 0.796–1.361; *p* = 0.770) LVEF had similar rates of long-term all-cause mortality (31.3% vs. 37.3% vs. 34.1%; log rank *p* ≥ 0.376 for all comparisons, respectively) (Fig. 2, left panel). Furthermore, development of LVEF had no significant impact on long-term HF-related rehospitalization (stable LVEF 21.1% vs. improved LVEF 24.1% vs. deteriorated LVEF 20.7%; log rank *p* ≥ 0.749 for all comparisons) (Fig. 2**, **right panel). Even at a shorter follow-up duration of 12 months, no significant differences between the stable, improved, and deteriorated LVEF groups were observed regarding all-cause mortality (21.1% vs. 24.1% vs. 23.0%) and HF-related rehospitalization (16.1% vs. 13.4% vs. 16.2%), respectively. Other secondary endpoints (e.g. in-hospital all-cause mortality, cardiac rehospitalization, MACCE at long-term follow-up) were also equally distributed among the three groups (Table 3). Even after excluding patients with a time interval of less than 1 month or more than 12 (Supplemental Fig. 1) or 24 months (Supplemental Fig. 2) between the prior and index LVEF assessment, similar rates of long-term all-cause mortality and long-term HF-related rehospitalization were observed in all three LVEF groups. The analysis of the prognostic endpoints of long-term all-cause mortality (log rank *p* ≥ 0.354 across groups) and HF-related rehospitalization (log rank *p* ≥ 0.374 across groups) in patients with stable, improved, and deteriorated LVEF stratified by the etiology of heart failure (i.e. ischemic and non-ischemic cardiomyopathy) also did not demonstrate significant differences between the three LVEF groups (Fig. 3).

**Table 3:** Follow-up data, primary and secondary endpoints

	Stable LVEF (*n* = 166)		Improved LVEF (*n* = 83)		Deteriorated LVEF (*n* = 440)		HR Improved vs. deteriorated	95% CI Improved vs. deteriorated	*p*-value Improved vs. deteriorated	HR Improved vs. stable	95% CI Improved vs. stable	*p*-value Improved vs. stable	*p*-value Stable vs. deteriorated
Primary endpoint, *n* (%)													
All-cause mortality, at 30 months	52	(31.3)	31	(37.3)	150	(34.1)	1.099	0.747–1.618	0.632	1.233	0.521–1.269	0.362	-
Secondary endpoints, *n* (%)	-												
All-cause mortality, in-hospital	5	(3.0)	1	(1.2)	20	(4.5)	0.256*	0.034–1.935*	0.093*	0.393*	0.045–3.417*	0.199*	-
All-cause mortality, at 12 months	35	(21.1)	20	(24.1)	101	(23.0)	1.049	0.649–1.695	0.844	1.167	0.673–2.020	0.583	-
Heart failure–related rehospitalization, at 12 months	26	(16.1)	11	(13.4)	68	(16.2)	0.817	0.432–1.543	0.533	0.813	0.402–1.645	0.565	-
Heart failure–related rehospitalization, at 30 months	34	(21.1)	19	(23.2)	87	(20.7)	1.085	0.661–1.783	0.747	1.058	0.603–1.855	0.845	-
Cardiac rehospitalization, at 30 months	46	(28.6)	24	(29.3)	123	(29.3)	0.975	0.629–1.508	0.908	0.991	0.605–1.623	0.971	-
Revascularization, at 30 months	12	(7.5)	7	(8.5)	29	(6.9)	1.227	0.537–2.801	0.628	1.135	0.447–2.882	0.790	-
Acute myocardial infarction, at 30 months	8	(5.0)	4	(4.9)	12	(2.9)	1.653	0.533–5.128	0.385	0.941	0.283–3.125	0.921	-
Stroke, at 30 months	3	(1.9)	4	(4.9)	13	(3.1)	1.529	0.499–4.695	0.457	2.571	0.575–11.494	0.217	-
MACCE, at 30 months	64	(38.6)	39	(47.0)	182	(41.4)	1.168	0.826–1.650	0.378	1.272	0.855–1.894	0.235	-
Follow-up data, median (IQR)													
Hospitalization time, days	9 (5–15)		11 (6–17)		8 (5–15)		-	-	0.222	-	-	0.533	0.690
ICU time, days	0 (0–0)		0 (0–0)		0 (0–1)		-	-	0.398	-	-	0.945	0.229
Follow-up time, days	920 (373–1718)		772 (387–1621)		837 (327–1390)		-	-	0.701	-	-	0.533	0.131

*CI*, confidence interval; *COPD*, chronic obstructive pulmonary disease; *HR*, hazard ratio; *ICU*, intensive care unit; *IQR*, interquartile range; *MACCE*, major adverse cardiac and cerebrovascular events. Level of significance *p* ≤ 0.05. Bold type indicates statistical significance. *An odds ratio with the corresponding 95% CI and *p*-value was provided for the endpoint of in-hospital mortality, accounting for the variance in hospitalization time

### Multivariate Cox regression analysis in the entire study cohort and within pre-specified subgroups

**Fig. 4:**
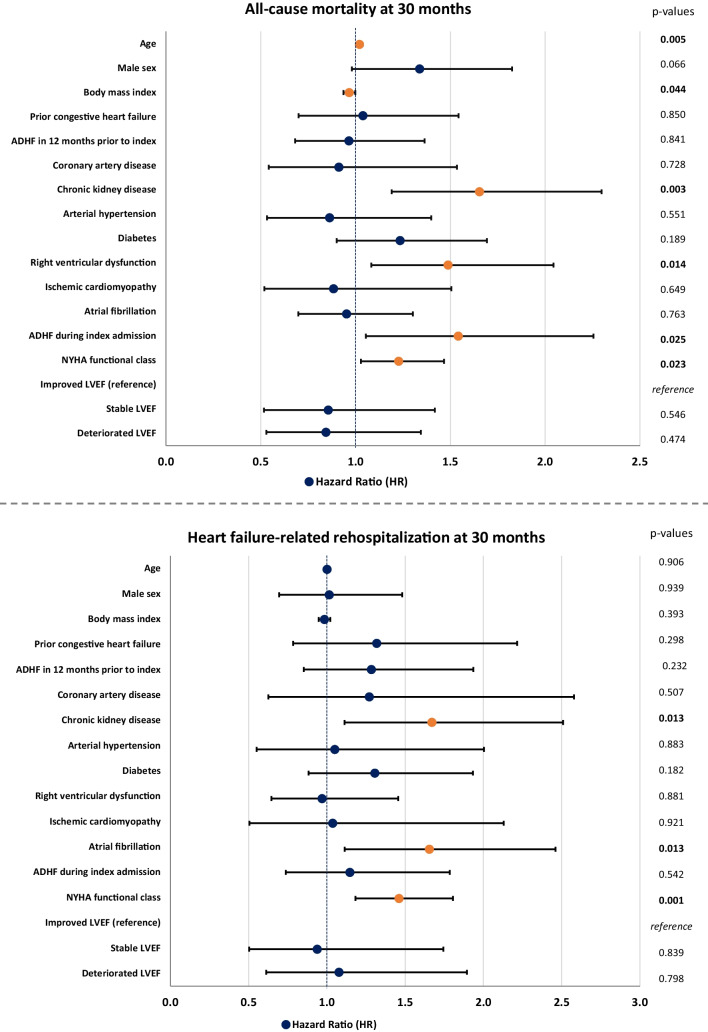
Forest plots demonstrating multivariable Cox regression analyses within the entire study cohort regarding the risk of all-cause mortality (upper panel) and heart failure–related rehospitalization (lower panel) at 30 months

**Fig. 5:**
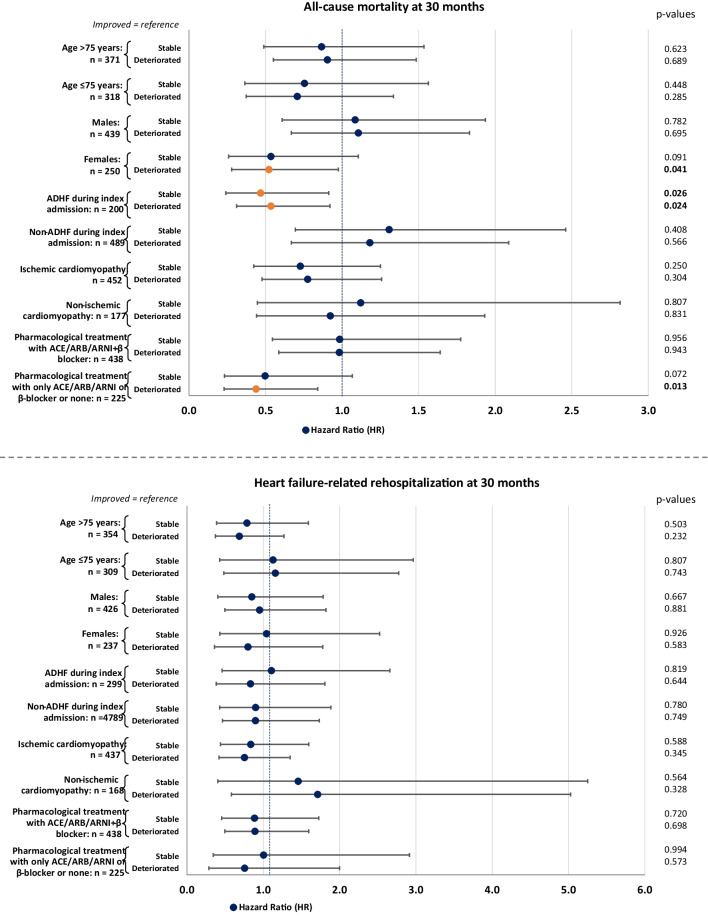
Forest plots demonstrating multivariable Cox regression analyses in pre-determined subgroups regarding the risk of long-term all-cause mortality (upper panel) and heart failure–related rehospitalization (lower panel). The multivariable Cox regression model was adjusted for age, sex, ICM, diabetes, and chronic kidney disease

After multivariable adjustment, the development of LVEF was still not associated with the risks of all-cause mortality and HF-related rehospitalization at long-term follow-up. However, age (HR = 1.021; 95% CI 1.006–1.037; *p* = 0.005), chronic kidney disease (CKD; HR = 1.654; 95% CI 1.190–2.298; *p* = 0.003), RVD (HR = 1.488; 95% CI 1.083–2.044; *p* = 0.014), ADHF during the index admission (HR = 1.542; 95% CI 1.055–2.255; *p* = 0.025), and NYHA functional class (HR = 1.228; 95% CI 1.028–1.467; *p* = 0.023) were identified as predictors of higher mortality, whereas increasing body mass index (BMI) was associated with a lower risk of mortality (HR = 0.967; 95% CI 0.937–0.999; *p* = 0.044) within the study cohort (Fig. 4, upper panel). Furthermore, CKD (HR = 1.671; 95% CI 1.113–2.509; *p* = 0.013), atrial fibrillation (HR = 1.655; 95% CI 1.114–2.460; *p* = 0.013), and NYHA functional class (HR = 1.461; 95% CI 1.182–1.806; *p* = 0.001) were associated with a higher risk for long-term HF-related rehospitalization in patients with HFmrEF (Fig. 4, lower panel). Using improved LVEF as the reference group, multivariable analyses in pre-specified subgroups defined by age, sex, ADHF, HF etiology, and medical therapy at discharge demonstrated significant differences in the prognostic impact of stable, improved, and deteriorated LVEF. Deteriorated LVEF was associated with significantly lower risk of mortality in females (HR = 0.522; 95% CI 0.279–0.975; *p* = 0.041) and in those not receiving combined angiotensin-converting enzyme inhibitor (ACEi)/angiotensin receptor blocker (ARB)/angiotensin receptor neprilysin inhibitor (ARNI) + β-blocker therapy (HR = 0.438; 95% CI 0.229–0.840; *p* = 0.013). In the subgroup of patients suffering from ADHF during the index admission, both stable (HR = 0.468; 95% CI 0.240–0.912; *p* = 0.026) and deteriorated LVEF (HR = 0.535; 95% CI 0.311–0.920; *p* = 0.024) were associated with lower long-term all-cause mortality compared to the improved LVEF group (Fig. 5, upper panel). However, the groups of stable, improved, and deteriorated LVEF were not associated with HF-related rehospitalization at long-term follow-up, even in the pre-determined subgroups (*p* ≥ 0.232 for all comparisons) (Fig. 5, lower panel).

### Distribution of LVEF-based HF categories and dynamic changes of LVEF in the follow-up period

**Fig. 6:**
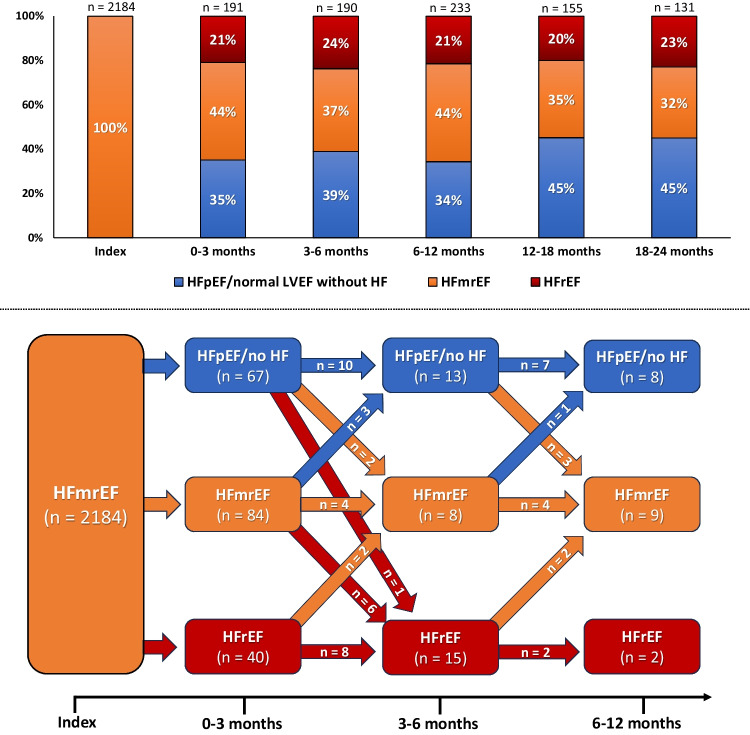
Bar charts displaying the distribution of LVEF-based HF categories (i.e. HFpEF/normal LVEF without HF, HFmrEF, and HFrEF) (upper panel) and a flow diagram demonstrating dynamic changes between HF categories during follow-up (lower panel)

LVEF remained a dynamic parameter in the follow-up period. Recurrent changes in the distribution of LVEF-based HF categories across 24 months of follow-up were observed. At any given time point during follow-up, 20–24%, 32–44%, and 34–45% of patients were categorized as HFrEF, HFmrEF, or HFpEF/normal LVEF without HF (Fig. 6, upper panel). In addition, transitions between HF categories observed in echocardiographic LVEF assessments in the 12 months following the index admission are displayed in the lower panel of Fig. 6. At last, available data on median LVEF and NT-proBNP levels obtained in clinical routine follow-up are displayed in Supplemental Fig. 3**.** Notably, a recurrent deterioration of LVEF at 24 and 30 months of follow-up was observed in patients initially assigned to the improved LVEF group. The difference in median LVEF reached statistical significance when comparing the improved with the deteriorated (*p* ≤ 0.047 for the comparison at 24 and 30 months), but not the stable LVEF group (*p* ≥ 0.080 for the comparison at 24 and 30 months) (Supplemental Fig. 3, left panel). No statistically significant differences regarding NT-proBNP levels (corrected for estimated glomerular filtration rate) during the follow-up period were observed between the groups of stable, improved, and deteriorated LVEF (*p* ≥ 0.103 for all comparisons) (Supplemental Fig. 3, right panel).

## Discussion

The present study investigates the prognostic impact of longitudinal changes of LVEF in a large retrospective cohort of patients hospitalized with HFmrEF. Through the comparison of prior LVEF and index LVEF, patients were stratified into three groups termed stable (i.e. prior LVEF of 41–49%), improved (i.e. prior LVEF ≤ 40% and an improvement ≥ 10% compared to the prior LVEF), and deteriorated LVEF (i.e. prior LVEF ≥ 50%). Longitudinal changes of LVEF were very common with only 24% of patients remaining in the category of HFmrEF at a median duration of 308 days between the prior and index echocardiography. However, prior LVEF was not associated with the primary endpoint of long-term all-cause mortality or with secondary endpoints including in-hospital or 12-month all-cause mortality as well as HF-related rehospitalization at 12 and 30 months. Analyses of LVEF assessments in the follow-up period demonstrated a persistently high dynamic of LVEF.

Since the new category of HF (i.e. HFmrEF) has been established in 2016, efforts have grown to characterize this cohort. Observational studies suggest that the median age and the proportion of women is higher in HFmrEF compared to HFrEF, but lower than in HFpEF [7, 29, 30]. In addition, prevalence of ischemic etiology seems to be similarly prevalent in HFrEF and HFmrEF and therefore more prevalent than in HFpEF [7, 30, 31]. Therefore, it has often been argued that HFmrEF could represent a transitional state between HFrEF and HFpEF, which are both characterized by more distinct pathophysiological properties [32, 33]. Accordingly, a large proportion of patients with HFmrEF transition to either HFrEF or HFpEF and vice versa, which was further confirmed by the main findings of the present study [15, 29, 34]. As displayed in Fig. 6, LVEF was also a highly dynamic parameter within our study cohort, with frequent transitions between LVEF-based HF categories observed in the time following the index admission. Even though LVEF is an established prognostic metric in HF, there is limited data on how prior LVEF impacts the prognosis of patients with HFmrEF. In a study by Savarese et al*.* [15] investigating the prognostic impact of longitudinal changes of LVEF across the spectrum of HF categories, 21% and 16% of patients previously classified as having HFpEF or HFrEF transitioned to HFmrEF, respectively [15]. In contrast, in the present study, 12% of patients with a prior LVEF ≤ 40% as well as an improvement of LVEF ≥ 10% and 64% with a prior LVEF ≥ 50% transitioned to HFmrEF. Especially the higher proportion of transitions from preserved LVEF could be explained by the fact that this group not only included patients with prior HFpEF but also patients with no established HF diagnosis before the index admission, whereas the study by Savarese et al*.* only included patients with established HF. This finding could suggest that, beyond patients transitioning from HFpEF to HFmrEF, there might be a large proportion of patients with incident HFmrEF. In general, Savarese et al*.* observed that an improvement in LVEF was associated with a more favourable prognosis, while deterioration of LVEF predicted adverse outcomes. However, this effect was more pronounced when comparing more extensive transitions between HFrEF and HFpEF rather than transitions to and from HFmrEF and — depending on the used comparator — changes to and from HFmrEF were not significantly associated with outcomes [15]. An association between transitions from HFpEF or HFrEF to HFmrEF and patient outcomes was not observed in the present study. A possible explanation for the absence of adverse prognosis in the deteriorated LVEF group of the present study could be explained by a significantly higher rate of hypertensive cardiomyopathy observed in this group, since previous research has indicated that hypertensive cardiomyopathy tends to have a more favourable prognosis compared to other HF etiologies [35–37]. In addition, improved LVEF was not associated with favourable outcomes. This finding was likely connected to the recurrent deterioration of LVEF observed in some patients of this cohort during the follow-up period (Supplementary Fig. 3, left panel). In general, the missing prognostic impact of the assigned LVEF groups might be explained by the dynamic nature of LVEF during the follow-up period. This dynamic could be particularly noteworthy in the cohort of HFmrEF due to its narrow EF interval (41–49%) and the presence of interrater variability in the assessment of LVEF. Considering the findings of previous studies investigating longitudinal changes of LVEF over time and our observations demonstrated in Fig. 6, it seems likely that a relevant proportion of patients will experience recurrent transitions between HF categories in the time following the index admission [29, 38]. As these changes could impact patients’ prognosis, prospective studies with pre-determined time intervals for LVEF assessment performed repeatedly by the same operators are required to further investigate the prognostic impact of longitudinal changes of LVEF, especially in patients with HFmrEF. At last, the equal proportion of patients experiencing ADHF in the index admission across all three LVEF groups should be considered as a noteworthy finding of the present study. Despite the distinct trajectories of LVEF development in the three groups, the event of ADHF occurred — at least in part — irrespective of longitudinal changes of LVEF. As ADHF itself is a prognostic marker in the disease course of HF, the similar rates of ADHF during the index admission and HF-related rehospitalization during the follow-up period could further explain the absence of prognostic differences between patients with stable, improved, and deteriorated LVEF.

Few studies [39, 40] have investigated the prognostic impact of transitions from reduced or preserved LVEF to the specific subgroup of HFmrEF and should therefore be acknowledged and discussed to provide a comprehensive overview of the current evidence. In a retrospective cohort study by Brann et al*.* [39] including 448 patients with HFmrEF, the deterioration of previous LVEF ≥ 50% compared to the improvement of previous LVEF ≤ 40% was associated with a significantly higher rate of both investigated composite endpoints (all-cause mortality or hospitalization and cardiovascular mortality or HF-related hospitalization) at a median follow-up of 2.24 years. However, analyses of the individual components of these composite endpoints did not demonstrate statistically significant differences (*p* ≥ 0.06 for all comparisons). Furthermore, the threshold for statistical significance was only met for all-cause mortality (*p* = 0.05) and all-cause hospitalization (*p* = 0.04) after multivariable adjustment, which could suggest an influence of other confounding factors. In contrast to our study, the deteriorated group was characterized by a significantly larger proportion of patients with malignancy (20%) compared to the improved (11%) and stable groups (5%) which showed significant impact on patient prognosis in univariate analysis. To address this, a separate analysis excluding patients with a history of malignancy and treatment with chemotherapy was performed, demonstrating that the composite endpoint of all-cause mortality and hospitalization no longer reached statistical significance. Additionally, the median time between the prior and index transthoracic echocardiography was around 1300 days and therefore significantly longer than in the present study (308 days), adding another level of diversity when comparing results [39]. Furthermore, Zhang et al*.* [40] investigated the prognostic impact of prior LVEF in a total of 1168 patients with HFmrEF hospitalized for ADHF at one institution. Higher risk for the composite of all-cause mortality and all-cause hospitalization was demonstrated when comparing the groups with improved and deteriorated LVEF, which was mainly driven by higher mortality. Besides other differences in baseline characteristics, significantly higher prevalence of malignancy was — once again — found in the deteriorated LVEF group. In contrast to our findings, there were higher rates of CAD within the deteriorated group, which could have impacted patients’ prognosis depending on the extent of previous therapeutic interventions. Unfortunately, no data regarding parameters such as prior PCI were provided to assess the impact of this difference [40]. Prescription of medical therapies (i.e. ACEi/ARB/ARNI, β-blockers, mineralocorticoid receptor antagonists (MRA), and statins) were relatively similar across all three groups when comparing data of the present study with data provided by Brann et al*.* and Zhang et al*.*, except for generally higher prescription rates of MRA in the study by Zhang et al*.* However, discrepancies regarding higher prescription rates of ACEi/ARB/ARNI, β-blockers, and MRA in the improved compared to the stable and deteriorated LVEF groups were more pronounced in their cohorts. In general, contemporary studies investigating the prognostic impact of prior LVEF in HFmrEF are characterized by varying study settings, heterogeneous cohort characteristics and differences in the definitions of endpoints. Therefore, the observed prognostic differences may be attributed to the presence or absence of confounding factors associated with LVEF that influence prognosis in patients with HFmrEF, regardless of the development of LVEF.

LVEF has been identified as a prognostic marker in previous studies and has been the cornerstone of defining inclusion criteria in many clinical trials [5–7, 10]. However, the classification of HF based predominantly on this parameter has been criticized for its inherent limitations [41, 42]. Firstly, measurement of LVEF through echocardiography is dependent on patients’ current condition (i.e. heart rate, bundle branch blocks, or pre- and afterload) and associated with high interobserver and intraobserver variability which can result in the misclassification of patients, especially considering the narrow LVEF range of HFmrEF [43–45]. This can be detrimental for patients because guideline recommendations for initiating cardioprotective pharmacotherapies and device therapies in HF are based on established LVEF thresholds [5, 6]. Secondly, it is debatable how accurately LVEF represents actual myocardial (dys)function, as normal values only indicate an appropriate ratio between stroke volume and left ventricular end-diastolic volume [46]. For example, studies have demonstrated that global longitudinal strain could be a stronger predictor of adverse clinical outcomes than LVEF, especially in cohorts with an LVEF > 35% [47]. Therefore, we believe the consideration of additional measures of myocardial form and function beside LVEF is necessary to provide more comprehensive medical management and improve the assessment of patients’ prognosis [48].

### Study limitations

Despite the efforts of statistical adjustment, the study results may be influenced by measured and unmeasured confounding due to the retrospective study design. Furthermore, only patients at our University Medical Centre were included in this study which limits generalizability to other patient cohorts. Specific causes of death beyond those occurring within the index hospitalization were not available for the present study since state resident registration offices are not allowed to disclose any personal information beyond the date of death due to German law. In addition, the use of medical therapies was not standardized, which could have impacted prognosis. Notably, echocardiographic assessments of prior LVEF were not obtained at pre-specified time intervals but were simply included based on availability. To obtain such a large patient cohort, echocardiographic data on prior LVEF was not only ascertained from our institution, but also from records of other hospitals or ambulatory cardiology visits stored in our electronic hospital information system. It is important to acknowledge that this approach might have introduced more heterogeneity and interobserver variability to the data and could have affected the precision of LVEF measurements.

### Conclusion

The present study supports the high frequency of transitions between HF categories (i.e. HFrEF, HFmrEF, and HFpEF) observed in previous observational studies. Transitions from prior LVEF values below or above the 41–49% threshold into the HFmrEF category were not associated with adverse outcomes in the follow-up period. These findings might be attributable to the dynamic nature of LVEF and the recurrent deterioration of LVEF in the improved group during the time of follow-up as well as the absence of additional prognostically negative factors in the deteriorated group, which often contributed to worse outcomes in prior studies.

## Supplementary Information

Below is the link to the electronic supplementary material.

Supplementary file1 Supplemental figure 1: Kaplan-Meier analyses comparing patients with stable, improved, and deteriorated LVEF regarding long-term all-cause mortality (left panel) and heart failure-related rehospitalization (right panel) within a select study cohort excluding patients with a minimum time interval of less than 1 month or more than 12 months between the prior and index LVEF assessment. (PPTX 92 KB)

Supplementary file2 Supplemental figure 2: Kaplan-Meier analyses comparing patients with stable, improved, and deteriorated LVEF regarding long-term all-cause mortality (left panel) and heart failure-related rehospitalization (right panel) within a select study cohort excluding patients with a minimum time interval of less than 1 month or more than 24 months between the prior and index LVEF assessment. (PPTX 94 KB)

Supplementary file3 Supplemental figure 3: Line graphs demonstrating changes of LVEF (left panel) and NT-proBNP levels (right panel) during the follow-up period among patients stratified by prior LVEF as stable, improved, or deteriorated LVEF. The data is presented as the median with the corresponding 25% and 75% percentiles. (PPTX 129 KB)

## Data Availability

The data underlying this article will be shared on reasonable request to the corresponding author.
