# Supplementary material for: Prognostic impact of prior LVEF in patients with heart failure with mildly reduced ejection fraction
Source: Clin Res Cardiol. 2024 Apr 15;114(5):570–88. doi: 10.1007/s00392-024-02443-0 (PMC12058930; doi:10.1007/s00392-024-02443-0)
Supplement: Supplementary file 1 — Supplementary file1 Supplemental figure 1: Kaplan-Meier analyses comparing patients with stable, improved, and deteriorated LVEF regarding long-term all-cause mortality (left panel) and heart failure-related rehospitalization (right panel) within a select study cohort excluding patients with a minimum time interval of less than 1 month or more than 12 months between the prior and index LVEF assessment. (PPTX 92 KB) [file 392_2024_2443_MOESM1_ESM.pptx]

## Slide 1
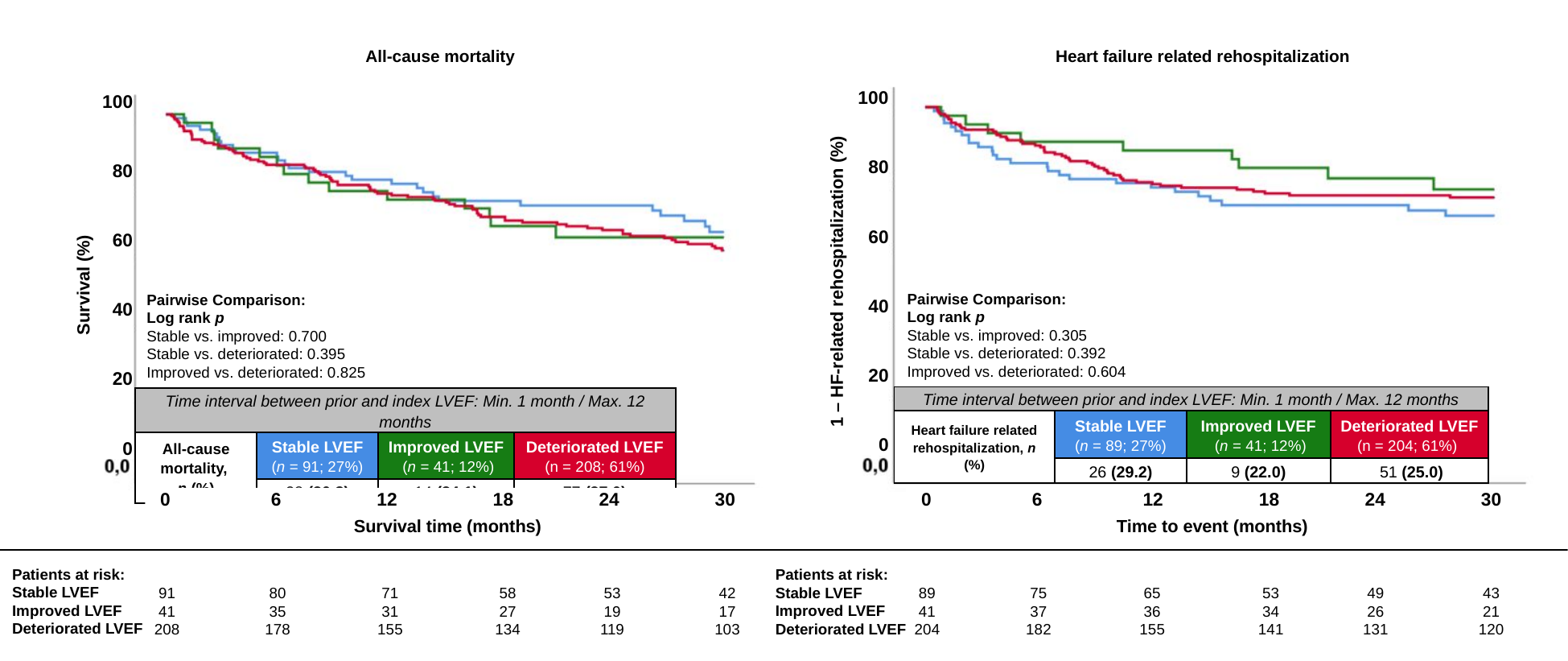

| All-cause mortality |
| --- |
| Heart failure related rehospitalization |
| --- |
100
80
60
40
20
0
100
80
60
40
20
0
1 – HF-related rehospitalization (%)
Survival (%)
Pairwise Comparison:
Log rank p
Stable vs. improved: 0.305
Stable vs. deteriorated: 0.392
Improved vs. deteriorated: 0.604
Pairwise Comparison:
Log rank p
Stable vs. improved: 0.700
Stable vs. deteriorated: 0.395
Improved vs. deteriorated: 0.825
| Time interval between prior and index LVEF: Min. 1 month / Max. 12 months | | | |
| --- | --- | --- | --- |
| Heart failure related rehospitalization, n (%) | Stable LVEF (n = 89; 27%) | Improved LVEF (n = 41; 12%) | Deteriorated LVEF (n = 204; 61%) |
| | 26 (29.2) | 9 (22.0) | 51 (25.0) |
| Time interval between prior and index LVEF: Min. 1 month / Max. 12 months | | | |
| --- | --- | --- | --- |
| All-cause mortality, n (%) | Stable LVEF (n = 91; 27%) | Improved LVEF (n = 41; 12%) | Deteriorated LVEF (n = 208; 61%) |
| | 28 (30.8) | 14 (34.1) | 77 (37.0) |
 0 6 12 18 24 30
 0 6 12 18 24 30
Survival time (months)
Time to event (months)
Patients at risk:
Stable LVEF
Improved LVEF
Deteriorated LVEF
Patients at risk:
Stable LVEF
Improved LVEF
Deteriorated LVEF
91
41
208
80
35
178
71
31
155
58
27
134
53
19
119
42
17
103
89
41
204
75
37
182
65
36
155
53
34
141
49
26
131
43
21
120
