# Supplementary material for: Prognostic impact of prior LVEF in patients with heart failure with mildly reduced ejection fraction
Source: Clin Res Cardiol. 2024 Apr 15;114(5):570–88. doi: 10.1007/s00392-024-02443-0 (PMC12058930; doi:10.1007/s00392-024-02443-0)
Supplement: Supplementary file 2 — Supplementary file2 Supplemental figure 2: Kaplan-Meier analyses comparing patients with stable, improved, and deteriorated LVEF regarding long-term all-cause mortality (left panel) and heart failure-related rehospitalization (right panel) within a select study cohort excluding patients with a minimum time interval of less than 1 month or more than 24 months between the prior and index LVEF assessment. (PPTX 94 KB) [file 392_2024_2443_MOESM2_ESM.pptx]

## Slide 1
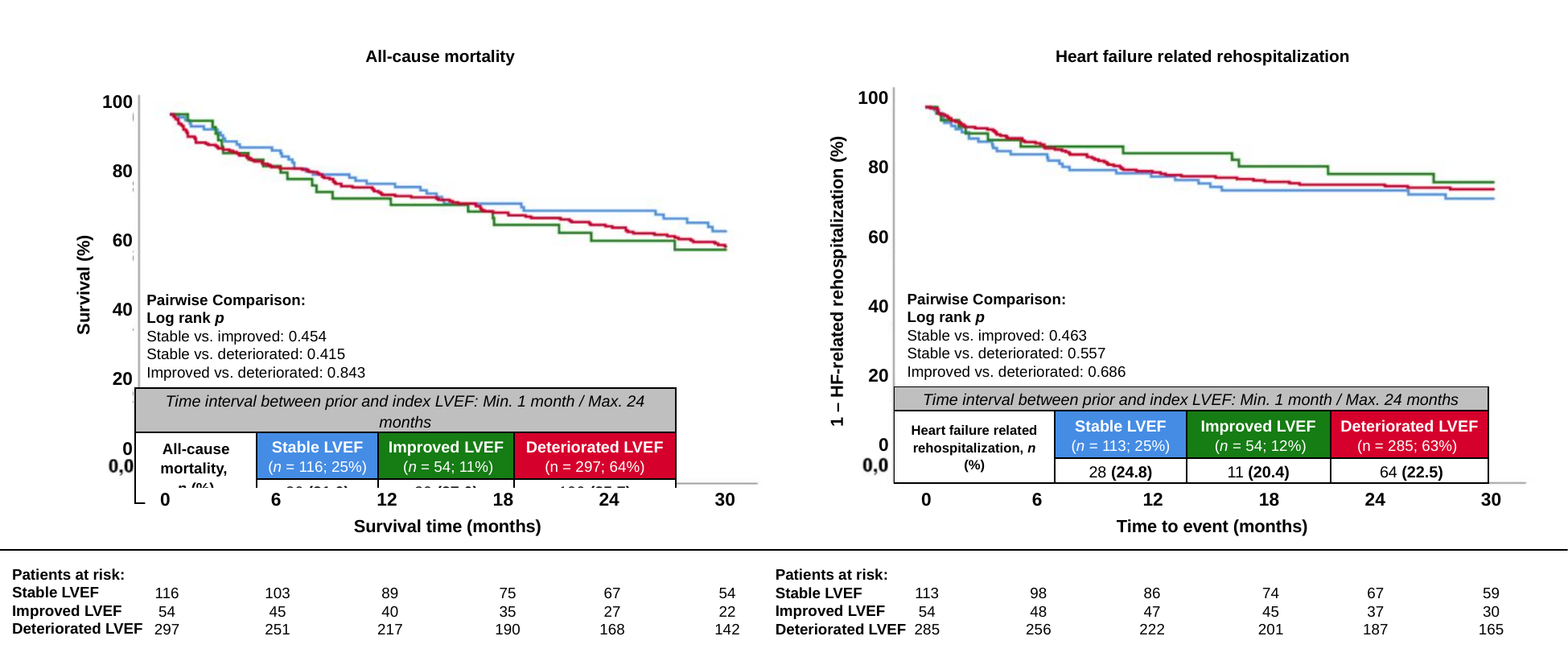

| All-cause mortality |
| --- |
| Heart failure related rehospitalization |
| --- |
100
80
60
40
20
0
100
80
60
40
20
0
1 – HF-related rehospitalization (%)
Survival (%)
Pairwise Comparison:
Log rank p
Stable vs. improved: 0.463
Stable vs. deteriorated: 0.557
Improved vs. deteriorated: 0.686
Pairwise Comparison:
Log rank p
Stable vs. improved: 0.454
Stable vs. deteriorated: 0.415
Improved vs. deteriorated: 0.843
| Time interval between prior and index LVEF: Min. 1 month / Max. 24 months | | | |
| --- | --- | --- | --- |
| Heart failure related rehospitalization, n (%) | Stable LVEF (n = 113; 25%) | Improved LVEF (n = 54; 12%) | Deteriorated LVEF (n = 285; 63%) |
| | 28 (24.8) | 11 (20.4) | 64 (22.5) |
| Time interval between prior and index LVEF: Min. 1 month / Max. 24 months | | | |
| --- | --- | --- | --- |
| All-cause mortality, n (%) | Stable LVEF (n = 116; 25%) | Improved LVEF (n = 54; 11%) | Deteriorated LVEF (n = 297; 64%) |
| | 36 (31.0) | 20 (37.0) | 106 (35.7) |
 0 6 12 18 24 30
 0 6 12 18 24 30
Survival time (months)
Time to event (months)
Patients at risk:
Stable LVEF
Improved LVEF
Deteriorated LVEF
Patients at risk:
Stable LVEF
Improved LVEF
Deteriorated LVEF
116
54
297
103
45
251
89
40
217
75
35
190
67
27
168
54
22
142
113
54
285
98
48
256
86
47
222
74
45
201
67
37
187
59
30
165
