# Supplementary figures and images for: Prognostic impact of prior LVEF in patients with heart failure with mildly reduced ejection fraction
Source: Clin Res Cardiol. 2024 Apr 15;114(5):570–88. doi: 10.1007/s00392-024-02443-0 (PMC12058930; doi:10.1007/s00392-024-02443-0)

## Slide 1
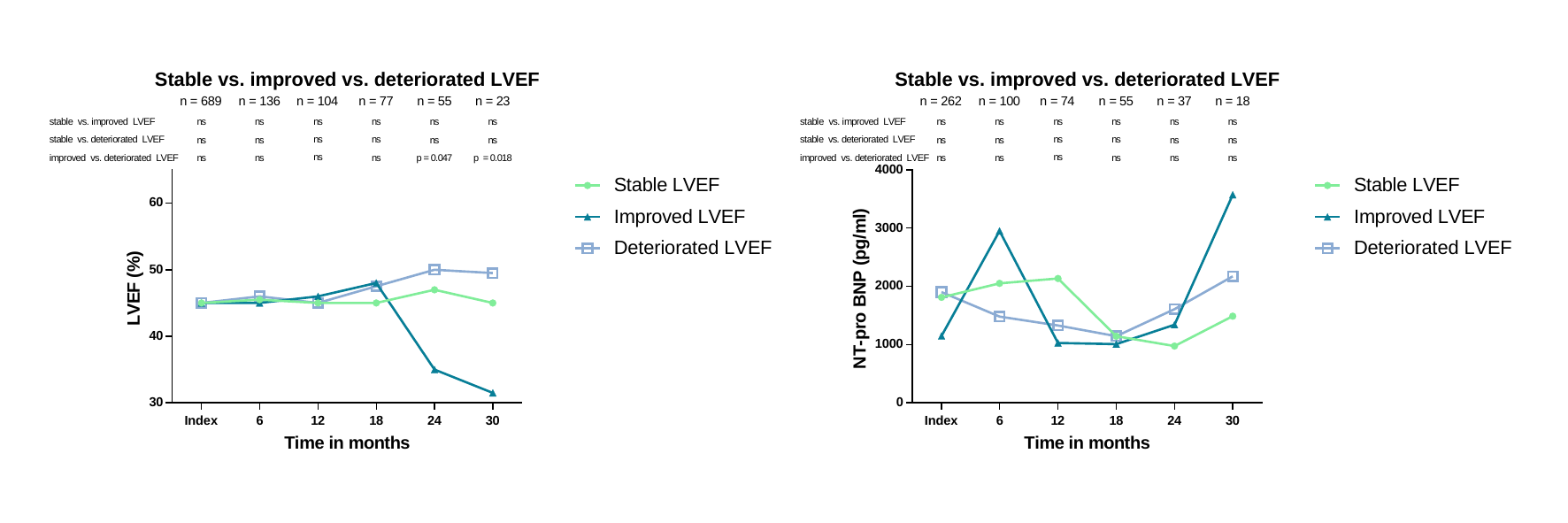

Index
Index

Supplement: Supplementary file 3 — Supplementary file3 Supplemental figure 3: Line graphs demonstrating changes of LVEF (left panel) and NT-proBNP levels (right panel) during the follow-up period among patients stratified by prior LVEF as stable, improved, or deteriorated LVEF. The data is presented as the median with the corresponding 25% and 75% percentiles. (PPTX 129 KB) [file 392_2024_2443_MOESM3_ESM.pptx]
